# Lipolytic actions of secretin in mouse adipocytes[S]

**DOI:** 10.1194/jlr.M038042

**Published:** 2014-02

**Authors:** Revathi Sekar, Billy K. C. Chow

**Affiliations:** School of Biological Sciences, University of Hong Kong, Pokfulam, Hong Kong

**Keywords:** lipolysis, secretin receptor, hormone sensitive lipase, protein kinase A

## Abstract

Secretin (Sct), a classical gut hormone, is now known to play pleiotropic functions in the body including osmoregulation, digestion, and feeding control. As Sct has long been implicated to regulate metabolism, in this report, we have investigated a potential lipolytic action of Sct. In our preliminary studies, both Sct levels in circulation and Sct receptor (SctR) transcripts in adipose tissue were upregulated during fasting, suggesting a potential physiological relevance of Sct in regulating lipolysis. Using SctR knockout and Sct knockout mice as controls, we show that Sct is able to stimulate lipolysis in vitro in isolated adipocytes dose- and time-dependently, as well as acute lipolysis in vivo. H-89, a protein kinase A (PKA) inhibitor, was found to attenuate lipolytic effects of 1 μM Sct in vitro, while a significant increase in PKA activity upon Sct injection was observed in the adipose tissue in vivo. Sct was also found to stimulate phosphorylation at 660^ser^ of hormone sensitive lipase (HSL) and to bring about the translocation of HSL from cytosol to the lipid droplet. In summary, our data demonstrate for the first time the in vivo and in vitro lipolytic effects of Sct, and that this function is mediated by PKA and HSL.

Lipolysis is a tightly regulated process involving enzymatic hydrolysis of stored triacylglycerol in adipocytes in response to physiological demands for maintaining body energy homeostasis. During times of energy depletion, lipolysis provides the needed free fatty acids (FFAs) as fuel for ATP production (1). For example, the energy for the continuous contractile activity of the heart muscle is met by the β-oxidation of long-chain FAs (2). FAs that are released from lipolysis are also involved in heat production through β-oxidation and mitochondrial uncoupling, leading to adaptive thermogenesis in brown adipose tissue (3). Dysregulation of lipolysis, such as impaired responsiveness to stimulated lipolysis and elevated circulating FFA levels could lead to lipotoxicity, which is associated with conditions such as obesity and insulin resistance (4, 5).

Among the hormones involved in the regulation of lipolysis, catecholamines and insulin are two well-recognized factors. Catecholamines via circulation or sympathetic innervations either stimulate lipolysis through the β-adrenergic receptor or exert anti-lipolytic activity by binding to the Gαi-coupled α-2 adrenergic receptor. On the other hand, insulin remains the most potent anti-lipolytic hormone by activation of phosphodiesterase 3B through the PI3K/Akt pathway (6). Other hormones such as glucocorticoids, glucagon, thyroid hormone, growth hormone, natriuretic peptide, and α-melanocyte stimulating hormone have also been shown to stimulate lipolysis. For instance, glucocorticoids activate lipolysis by downregulating phosphodiesterase 3B and perilipin, and upregulating adipose triglyceride lipase (ATGL) (7). TNF-α, a cytokine, downregulates perilipin thereby bringing about lipolysis. In addition to protein kinase A (PKA)-dependent lipolysis, other kinases such as ERK1/2 through the PKC/MAPK pathway and AMP-activated protein kinase as well as cGMP-dependent kinase are also involved in regulating lipolysis (6, 8, 9). With new discoveries on the molecular mechanisms of lipolysis, some of the proteins involved in its metabolic pathway have been proposed as drug targets for metabolic disorders (10).

Secretin (Sct) is best known for its action in stimulating bicarbonate release from pancreatic ductal epithelial cells and has been thoroughly studied for its gastrointestinal functions (11, 12). More recently, the role of Sct as a neuroactive peptide has been substantiated (11, 13–15) and it has been found to regulate, at multiple levels, in osmoregulation (16–19). Although Sct has recently been shown to be an anorectic peptide (20, 21), its metabolic role in regulating lipolysis remains a controversial issue. There is evidence supporting (22–25) and some negating (26–28) the lipolytic effects of Sct. Besides, there was no information as yet on the underlying cellular mechanism, secondary messenger pathway, and mode of action on the actions of Sct on adipocytes. In this report, using secretin receptor knockout (SctR^−/−^) and secretin knockout (Sct^−/−^) mice as controls in comparison with wild-type (Wt) animals, we sought to study the function of Sct in lipolysis and investigate the molecular mechanisms involved in this process in mouse adipocytes.

## MATERIALS AND METHODS

### Reagents

Antibodies for hormone sensitive lipase (HSL), HSL-660^ser^, HSL-563^ser^, HSL-565^ser^, perilipin, ATGL, and GAPDH were purchased from Cell Signaling Technology (Beverly, MA); the antibody for ABDH5 was from Abcam (Cambridge, MA), G0S2 from LifeSpan BioSciences (Seattle, WA), and perilipin-522^ser^ from Vala Sciences (San Diego, CA). The β_3_-specific adrenergic receptor (β_3_-AR) agonist, CL-316243, was from Sigma (St. Louis, MO). HSL inhibitor CAY10499 (CAY) was from Cayman Chemical (Ann Arbor, MI). The enzyme immunoassay (EIA) kit for Sct was purchased from Phoenix Pharmaceuticals, Inc. (Burlingame, CA). Glycerol and FFA kits were from BioAssay Systems (Hayward, CA). Other reagents were from Sigma (St. Louis, MO).

### Animals

Procedures of animal care and handling were in accordance with the protocols approved by the Committee on the Use of Live Animals in Teaching and Research of the University of Hong Kong. All experiments were carried out using adult mice (23–26 g) of at least N5 generation, which were kept in a temperature-controlled room with a 12 h light-dark cycle. Mice were fed ad libitum with standard rodent chow (#5010; Test Diet, Richmond, IN) and water unless otherwise stated.

### Fasting experiments

Age- and weight-matched mice (8–9 weeks old) were used for all the experiments. Food was removed from a cohort of mice at the beginning of the dark cycle and epididymal fat pads were collected from fasted and control mice at 0, 12, 18, and 24 h, and were immediately snap-frozen with liquid nitrogen for real-time quantitative measurements of secretin receptor (SctR) transcripts. Blood was collected from mice at the same time points and plasma Sct concentrations were measured by enzyme immunoassays (Phoenix Pharmaceuticals Inc.).

### Adipocyte isolation and in vitro lipolysis assay

Adipocyte isolation and in vitro lipolysis assay were done as described (29, 30), with minor modifications. Briefly, epididymal fat pads from Wt, SctR^−/−^, and Sct^−/−^ mice were placed in Krebs-Ringer bicarbonate buffer with 30 mM HEPES (KRBH buffer) supplemented with 3% FA-free BSA, 500 nmol/l adenosine, and 1 mg/ml collagenase type I (Worthington Biochemical, Lakewood, NJ) and agitated for 60 min at 37°C. The cells were washed three times and then resuspended in KRBH buffer with 3% FA-free BSA. Suspended adipocytes (10^5^ cells/ml) were used for a lipolysis assay by incubating them with or without Sct (1 pM to 1 μM) in the presence or absence of a nonselective β-adrenergic agonist, isoproterenol (Iso) (1 μM) or β_3_-AR-specific agonist CL-316243 (5 μM). After 60 min, glycerol release was measured with a kit from BioAssay Systems. For pathway analysis, cells were pretreated with 10 μM H-89 (PKA inhibitor), 1 μM R0-31-8220 (PKC inhibitor), 10 μM SP 600125 (JNK inhibitor), or 10 μM or 1 μM CAY (HSL inhibitor) in the presence or absence of 1 μM Sct. For time-dependent effects, cells were treated with or without 1 μM Sct, and glycerol release was measured 0, 10, 20, 30, 40, 50, 60, and 90 min after stimulation.

### Serum analysis

For in vivo lipolysis assay, baseline serum samples were collected from 18 h-fasted Wt, SctR^−/−^, and Sct^−/−^ mice by blood drawn from the tail vein. These mice were then treated with an intra-peritoneal (ip) injection of either Sct (0.5 mg/kg) or CL-316243 (0.1 mg/kg), and serum samples were collected after 15 min. For time point analysis, serum was collected at 5, 10, 15, 20, 30, and 45 min after ip injection of Sct (0.5 mg/kg). FFA levels were determined by using a kit from BioAssay Systems.

### PKA assay

A PepTag PKA activity assay kit (Promega) was used. Mice were fasted for 18 h and ip injected with either PBS or Sct (0.5 mg/kg). Fifteen minutes after ip injection, epididymal fat pads were removed and processed as per the manufacturer's instructions.

### Quantitative real-time PCR

Total RNA from epididymal fat tissue was extracted using Trizol reagent (Invitrogen). Total RNA (1 μg) was used for the synthesis of first strand cDNA. The cDNA was diluted 3-fold and real-time PCR was performed either with specific TaqMan probes (GAPDH, 4352339E; SctR, Mm01290790_m1) or with a SYBR PCR Master Mix kit (Applied Biosystems) and primers (sequences listed in **Table 1**). The specificity of the SYBR Green PCR signal measured by the 7300 real-time PCR system (Applied Biosystems) was confirmed by melting curve analysis and agarose gel electrophoresis. The threshold cycle (*C_t_*) value was used for calculating the ratio change in the target gene relative to the GAPDH control gene which was determined by the 2^−ΔΔ^*Ct* method (31).

**TABLE 1.** Sequence of the oligonucleotides used for real time PCR

Primer	Sequence 5′ → 3′
HSL	CAACATGGCATCAACCACTGGCCTGGGATCAGAGGTGATG
ATGL	TGTGGCCTCATTCCTCCTACTCGTGGATGTTGGTGGAGCT
β_3_-AR	GAGCCAGTGGTGGCGTGTAGGACAGCAGCGATTGGAGT
GAPDH	TGTGTCCGTCGTGGATCTGACCTGCTTCACCACCTTCTTGAT
CD 36	GCCAAGCTATTGCGACATGAATCTCAATGTCCGAGACTTTTCAAC
LPL	GGGAGTTTGGCTCCAGAGTTTTGTGTCTTCAGGGGTCCTTAG
aP2	TGGAAGCTTGTCTCCAGTGAAATCCCCATTTACGCTGATG
Adiponectin	CAGGCATCCCAGGACATCCCCAAGAAGACCTGCATCTCCTTT

aP2, adipocyte protein2.

### Western blot analysis

Mice were treated with an ip injection of either PBS or Sct (0.5 mg/kg) and 15 min later epididymal fat depots (100 mg) were subsequently homogenized in a buffer composed of 25 mM Tris-HCl (pH 7.4), 25 mM NaCl, 1 mM MgCl_2_, 2.7 mM KCl, and protease and phosphatase inhibitors (0.5 mM Na_3_VO_4_, 1 mM NaF, 1 μM leupeptin, 1 μM pepstatin, 1 μM okadaic acid, and 0.1 mM PMSF). For isolated adipocytes, cells were stimulated with or without Sct (1 μM) or Iso (1 μM) for 30 min before being lysed with buffer as described above. Western blot analysis of the lysates was performed as described (32) with dilutions of primary antibodies as suggested by their respective companies.

### Continuous Sct infusion

Adult male mice (8–9 weeks old) were treated with PBS or Sct [2.5 nmol/kg/day, a dose similar to that published previously (33)] by ip implantation of Alzet^®^ osmotic minipumps (Alzet, Cupertino, CA) as described (16). Blood samples were collected from the tail vein at 0, 18, and 24 h for measurements of FFAs (BioAssay Systems). Epididymal fat pads were removed after 24 h and snap-frozen in liquid nitrogen for subsequent real-time PCR analysis.

### Immunofluorescence imaging and immunohistochemical staining

Immunocytochemical staining was performed as described (34) with minor modifications. Isolated adipocytes were treated with or without 1 μM Sct or 1 μM Iso for 20 min, fixed, and then blocked for 1 h in 5% goat serum at room temperature. Cells were then incubated overnight at 4°C with the primary antibody, anti-HSL (Cell Signaling Technology) (1:50 dilution). This was followed by a wash and 1 h incubation at room temperature with Alexa Fluor 488 goat anti-rabbit secondary antibody (1:300 dilution; Molecular Probes). After washing, the cells were placed in SlowFade Gold antifade reagent (Invitrogen) and confocal images were obtained using a Carl Zeiss LSM 710-NLO (Carl Zeiss, Oberkochen, Germany). For immunohistochemical staining, epididymal adipose tissue was fixed in 3.7% formalin, embedded in paraffin, and sectioned (7 μm). Immunostaining was performed with a Leica Bond-Max automatic immunostainer (Leica, Bannockburn, IL) according to the recommended procedure using a rabbit anti-mouse SctR antibody [1:300; raised in our laboratory using a synthetic peptide (R-A-E-C-L-R-E-L-S-E-E-K-K) that is present in the mouse SctR] (17, 35).

### Statistical analysis

All data are shown as means ± SE. The deviations between groups were analyzed using Prism 3.0 software (GraphPad Software Inc., San Diego, CA). An unpaired *t*-test was performed when two groups were under consideration, whereas data from >2 groups were analyzed by one-way ANOVA, followed by Dunnett's test.

## RESULTS

### Sct level in circulation and SctR expression in epididymal adipose tissue are increased during starvation

**Fig. 1.**
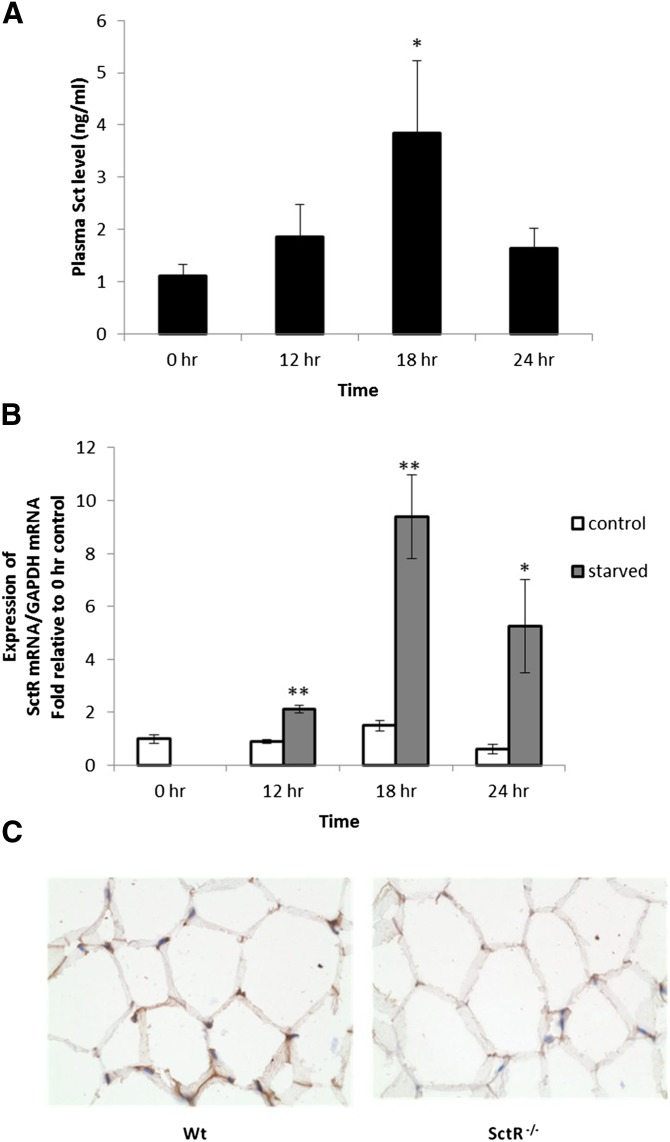
Increases of both the Sct peptide level in circulation and the SctR transcript level in epididymal fat tissue during starvation. Mice were starved and blood samples or fat tissues were obtained for measurements of Sct peptide and SctR transcript levels, respectively, at specified hours. A: Sct peptide concentrations in the circulation measured by an EIA kit were found to be significantly increased after starvation. B: SctR transcript levels measured by real-time PCR were found to be significantly increased after starvation. For (A) and (B), n = 9–11 (*P < 0.05, **P < 0.0005 versus 0 h): C: SctR protein was localized on the adipose cell membrane of Wt mice by immunohistochemical staining using SctR^−/−^ mice as negative controls.

To determine whether Sct plays a physiological role in lipid mobilization, we first studied the effects of fasting on the Sct level in the circulation and SctR expression in the epididymal adipose tissue of mice. Interestingly, we found that the Sct level in the circulation (**Fig. 1A**) and SctR (Fig. 1B) transcript in the adipose tissue increased in a time-dependent manner, and the most significant increases were observed after 18 h fasting (3.2 ± 1.1-fold and 9.4 ± 1.6-fold increase in Sct peptide in blood and SctR transcript in adipose tissue, respectively). Expression of SctR protein in the adipocyte cell membrane was confirmed by immunohistochemical staining (Fig. 1C) using the same tissue from SctR^−/−^ mice as a negative control. In summary, the increase of both the ligand in the circulation and the receptor in the fat tissue suggests potential activity of Sct in fat metabolism in response to starvation.

### Sct stimulates lipolysis in isolated adipocytes from mice via SctR and PKA

**Fig. 2.**
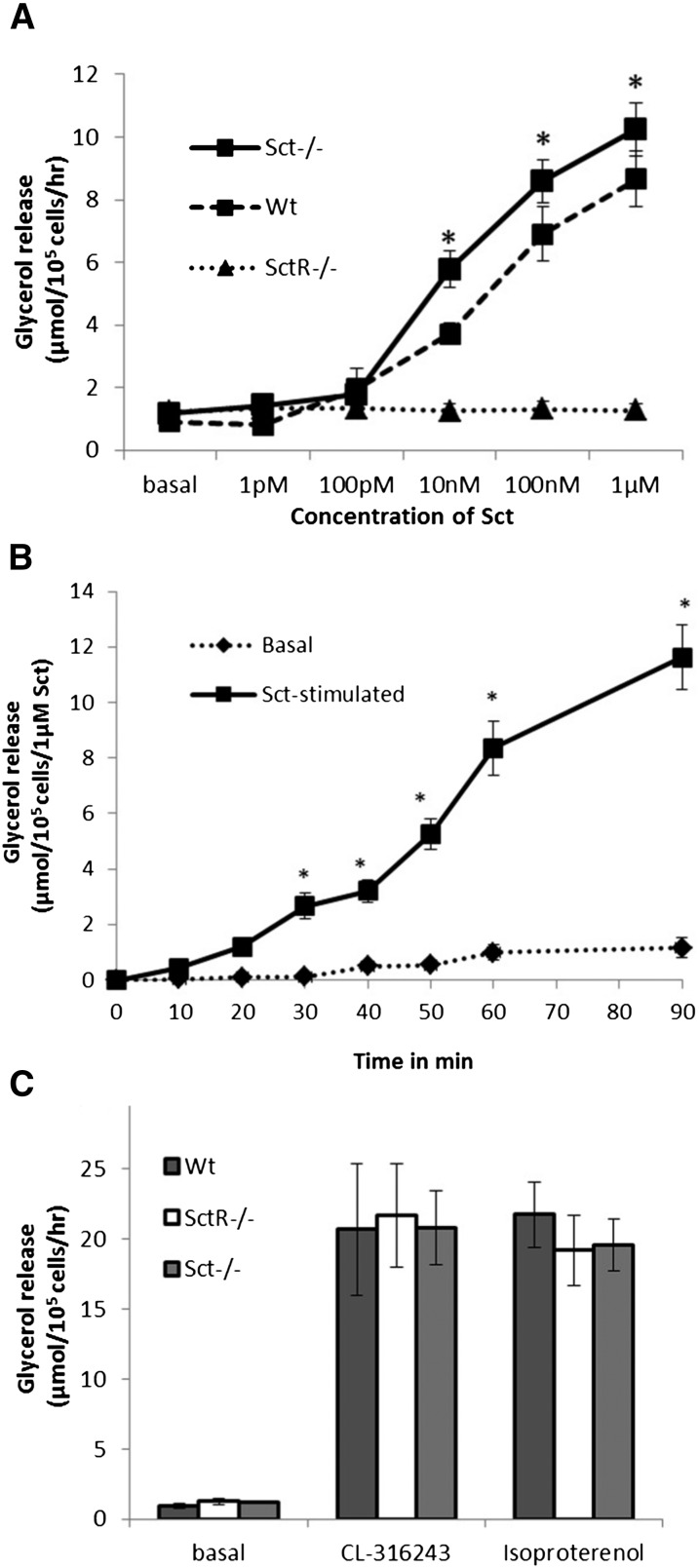
Sct via SctR stimulates lipolysis in dose- and time-dependant manners. A: Glycerol release is increased in the presence of graded concentrations of Sct treatment for 1 h in Wt and Sct^−/−^ adipocytes but not in SctR^−/−^ adipocytes, indicating specificities of the lipolytic actions of Sct. B: Sct (1 μM) stimulates glycerol release time-dependently in Wt adipocytes. C: Adipocytes from Wt, Sct^−/−^, and SctR^−/−^ mice were found to exhibit similar basal lipolytic activities and responded similarly to 1 μM Iso or 5 μM CL-316243 treatment for 1 h. Data are means ± SEM of three separate experiments performed in triplicate. *P < 0.0001.

To determine whether Sct stimulates lipolysis, we investigated the in vitro rate of lipolysis in the presence of graded concentrations of Sct in isolated adipocytes in Wt mice, using SctR^−/−^ and Sct^−/−^ as negative and positive controls, respectively. In this study, the rate of glycerol release from isolated adipocytes into the culture medium was determined to reflect its lipolytic activity. Sct was able to stimulate a dose-dependent release of glycerol in Wt adipocytes (**Fig. 2A**) with an EC_50_ of 37.5 nM. This lipolytic response of Sct was found to be specific to its receptor, as this effect was completely abolished in SctR^−/−^ adipocytes (Fig. 2A), but could be reproduced in Sct^−/−^ adipocytes (Fig. 2A). Sct also stimulated lipolysis in Wt adipocytes in a time-dependent manner (Fig. 2B). Isolated adipocytes from Wt, Sct^−/−^, and SctR^−/−^ mice had similar basal lipolytic activity (glycerol release in μmol/10^5^ cells/h), as well as no significant differences in their lipolytic responses to 1 μM Iso (a nonselective β-adrenergic agonist) or 5 μM CL-316243 (β_3_-AR-specific agonist) (Fig. 2C), indicating that SctR^−/−^ adipocytes are able to respond normally to other lipolytic agents.

**Fig. 3.**
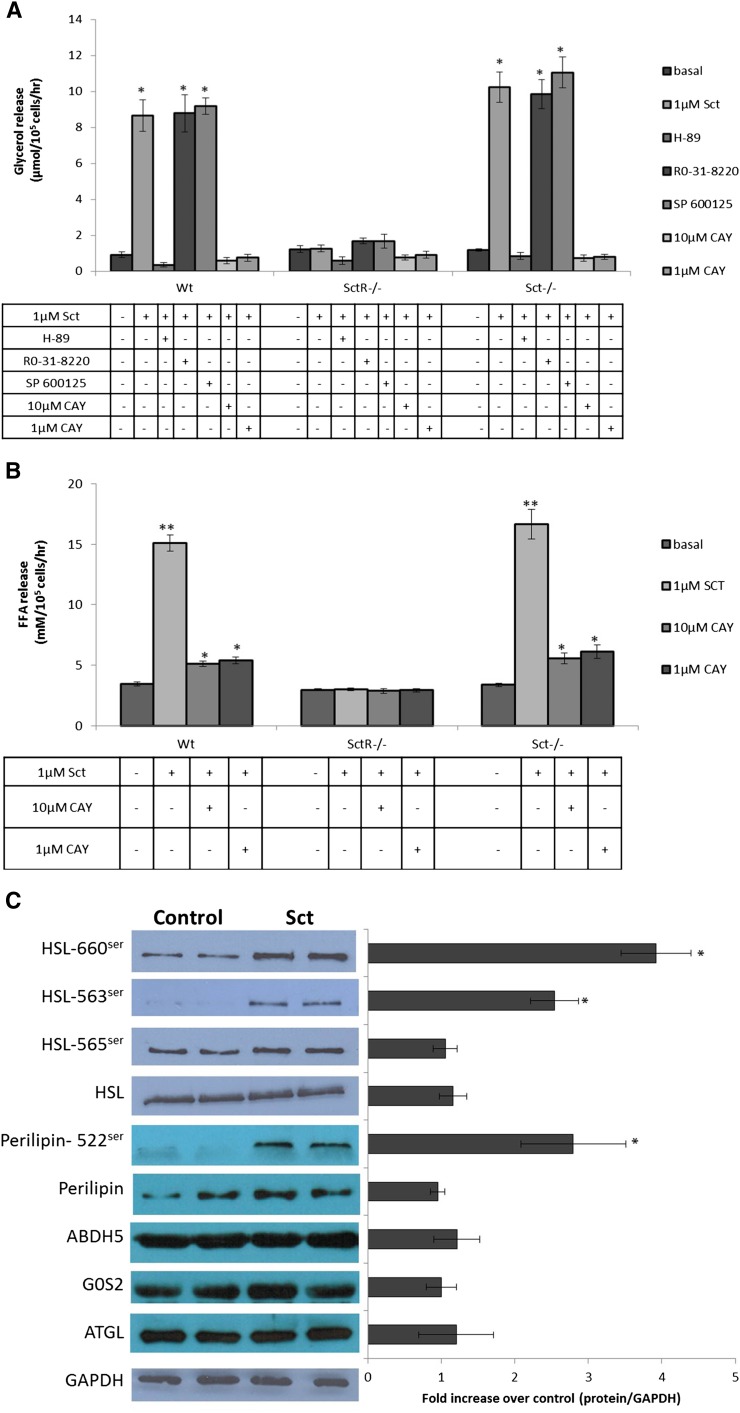
Lipolytic actions of Sct are mediated by PKA and by phosphorylation of 660^ser^ and 563^ser^ of HSL in vitro. A: Isolated adipocytes were treated with 10 μM H-89 (PKA inhibitor), 1 μM Ro-31-8220 (PKC inhibitor), 10 μM SP 600125 (JNK inhibitor), or 10 μM or 1 μM CAY (HSL inhibitor) before stimulation with 1 μM Sct for 1 h. Lipolysis by Sct in Wt and Sct^−/−^ adipocytes was completely abrogated by H-89, but no effects were observed for Ro-31-8220 and SP 600125. SctR^−/−^ serves as the negative control. Data are means ± SEM of three separate experiments performed in triplicate. *P < 0.0001 from basal. B: Small amounts of FFA release are still observed in adipose cells incubated with 10 μM or 1 μM CAY (HSL inhibitor) along with 1 μM Sct for 1 h. **P < 0.0001, *P < 0.001 from basal. C: Isolated adipocytes incubated with 1 μM Sct for 1 h were used for Western analysis. Sct phosphorylates HSL at 660^ser^ and 563^ser^, but not at 565^ser^, and phosphorylates perilipin at 522^ser^ while total HSL, ATGL, G0S2, ABDH5, perilipin, and GAPDH proteins remain unaltered by Sct. The left-hand chart represents fold changes compared with control by densitometric analysis. Data are means ± SEM of three separate experiments performed in duplicate. *P < 0.05 from control.

After showing the lipolytic actions of Sct, we next investigated its secondary messenger pathway. In this study, the specific inhibitor of PKA (10 μM H-89), PKC (1 μM Ro-31-8220), or JNK (10 μM SP 600125) was preincubated with adipocytes before stimulation by 1 μM Sct; it was found that only H-89, but not R0-31-8220 or SP 600125, could completely abolish lipolytic actions of Sct in Wt and Sct^−/−^ adipocytes (**Fig. 3A**). The rates of glycerol release in SctR^−/−^ adipocytes were similar to basal levels in all treatment groups due to the lack of SctR in these cells. Taken together, our data show that the lipolytic effect of Sct is mediated by the PKA pathway. Additionally, incubation with the HSL specific inhibitor, 10 μM or 1 μM CAY, completely abolished the glycerol release stimulated by 1 μM Sct (Fig. 3A). Furthermore, FFA release by 1 μM Sct was attenuated to around 33–35% on coincubation with 10 μM or 1 μM CAY (Fig. 3B). Such a response is similar to that of catecholamine in HSL-null mouse adipocytes, wherein catecholamine stimulation completely abolished glycerol release and severely reduced FFA release (36, 37). This study suggests that HSL is the primary downstream enzyme involved, while delineating the role for other enzymes to be minimal or none.

### Sct activates phosphorylation of 660^ser^ and 563^ser^ in HSL and translocation of HSL from cytosol to lipid droplets in adipocytes

To confirm the involvement of HSL as a downstream regulator, we measured changes in the phosphorylation of the serine residues at positions 660, 563, and 565 of HSL after Sct stimulation. While the protein levels of GAPDH and total HSL of the Wt adipocytes were similar with or without Sct stimulation (Fig. 3C), Sct incubation led to a 4.26 ± 0.75-fold (*P* < 0.05) and a 1.99 ± 0.32-fold (*P* < 0.05) increase in phosphorylation at positions 660 and 563, respectively, but not Ser^565^ (Fig. 3C). Perilipin phosphorylation is essential for HSL-stimulated lipolysis, and Sct consistently stimulated phosphorylation of perilipin (Fig. 3C). Additionally, stimulation with Sct did not have any effect on the triglyceride lipase, ATGL, and its positive and negative regulators ABDH5 and G0S2, respectively (Fig. 3C), thus confirming that HSL is the primary enzyme involved in the lipolytic effect of Sct. In summary, our findings suggest that Sct-activated lipolysis in adipocytes could be mediated by the phosphorylation of HSL at 660^ser^ and 563^ser^.

**Fig. 4.**
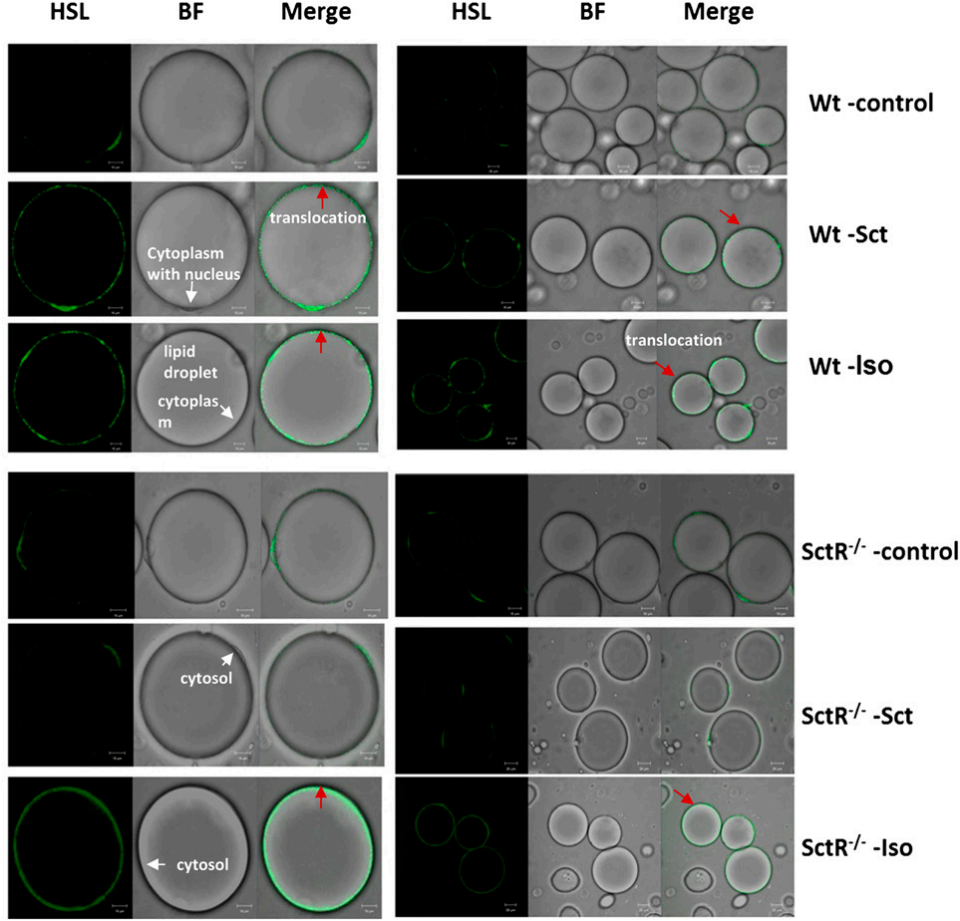
Sct stimulates translocation of HSL from cytosol to lipid droplet. Isolated adipocytes from Wt or SctR^−/−^ mice were stimulated with or without 1 μM Sct or 1 μM Iso (positive control). Cells were fixed and translocation of HSL from the cytosol to lipid droplet was visualized by confocal microscopy using an anti-HSL antibody (1:50 dilution) and subsequent incubation with Alexa-488 sary antibodies (1:300 dilution). Red arrows in the figure denote the translocation process. BF, bright field.

To understand further the cellular mechanism of Sct on lipolysis, translocation of HSL from the cytosol to the lipid droplet in isolated adipocytes was studied. Through immunofluorescent imaging, in the positive control, treatment of isolated adipocytes with Iso clearly resulted in the translocation of HSL from the cytosol to the phospholipid monolayer surface of the lipid droplet in Wt and SctR adipocytes (**Fig. 4**). This translocation of HSL was also observed in Sct-treated Wt cells, but not in Sct-treated SctR^−/−^ adipocytes or in unstimulated Wt and SctR^−/−^ cells (Fig. 4). Our findings suggest that the lipolytic action of Sct is mediated via the translocation of HSL from the cytosol to the lipid droplet.

### Sct induces an acute lipolytic response through SctR and PKA in vivo

**Fig. 5.**
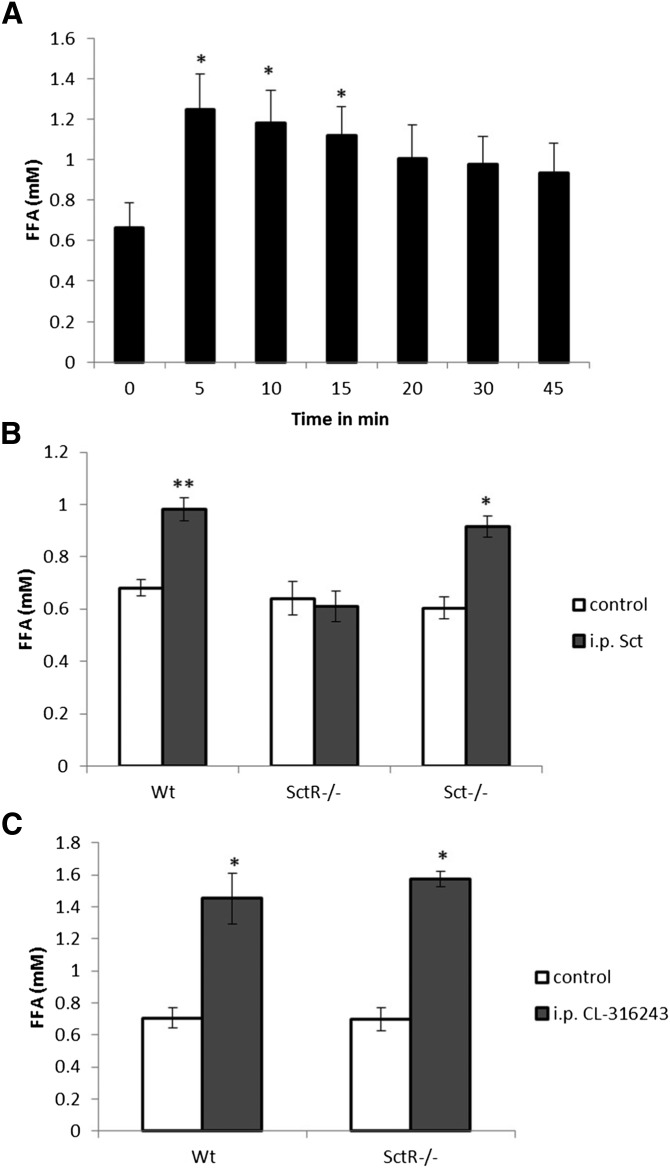
Sct stimulates an acute FFA release in vivo through SctR. Stimulation of lipolysis by Sct was detected by measuring changes in the levels of FFAs in circulation after ip injection of Sct. Blood was collected from the tail vein for measurement of FFAs. A: Sct (0.5 mg/kg, ip) induced an acute lipolytic response in Wt mice (n = 9) up to 15 min after injection. *P < 0.05. B: The same dose of ip Sct significantly augments FFA concentrations in Wt (n = 8) and Sct^−/−^ (n = 9) mice, but not in SctR^−/−^ (n = 7) mice. *P < 0.0005, **P < 0.0001 from control. C: CL-316243 (0.1 mg/kg), a β_3_-AR agonist, had similar effects in Wt and SctR^−/−^ mice (n = 8 each). *P < 0.005 from control.

After showing the in vitro lipolytic actions of Sct through PKA and HSL in isolated adipocytes, our next objective was to investigate whether Sct could induce lipolysis in vivo. After 5, 10, and 15 min ip injections of Sct (0.5 mg/kg), we found significant elevation of circulating FFAs in the blood (**Fig. 5A**). This lipolytic effect of Sct is specific to SctR because the rise in the FFA level was not observed in SctR^−/−^, while it could be reproduced in Sct^−/−^ mice (Fig. 5B). In addition, both the Wt and SctR^−/−^ mice showed a normal lipolytic response to ip CL-316243 (0.1 mg/kg) as controls (Fig. 5C), indicating the normal functioning of these animals in response to the β_3_-AR-specific agonist.

**Fig. 6.**
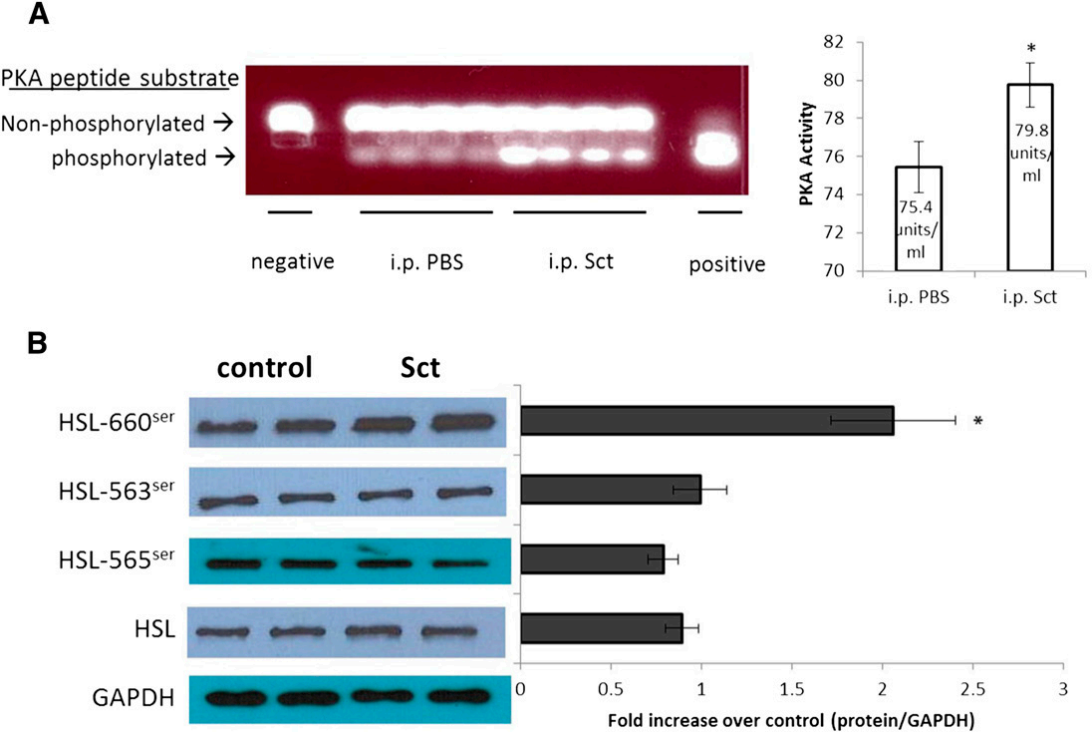
In vivo Sct activates PKA and phosphorylates HSL at serine residue 660^ser^. Mice were ip injected with Sct (0.5 mg/kg in PBS) or PBS, and epididymal adipose tissue was collected 15 min after injection for PKA assay and Western blotting. A: Sct stimulates PKA activity; the left-hand panel represents spectophotometric measurements of the bands. *P < 0.05. B: Sct stimulates phosphorylation of HSL-660^ser^ and does not affect the protein levels of HSL-563^ser^, HSL-565^ser^, and total HSL. The left-hand chart represents fold changes compared with control by densitometric analysis. Data are means ± SEM of three separate experiments performed in duplicate. *P < 0.05.

To test the in vivo involvement of PKA and HSL in carrying out the lipolytic effects of Sct, mice were ip injected with Sct (0.5 mg/kg) or control PBS, and epididymal adipose tissue was removed 15 min after for PKA measurements. It was found that PKA activity in epididymal adipose tissue was significantly upregulated in Sct-injected mice when compared with control mice (**Fig. 6A**). Similar to the in vitro studies, Sct injection resulted in increased phosphorylation of 660^ser^ in HSL (Fig. 6B), while there were no significant changes in the phosphorylation status of 563^ser^ and 565^ser^, as well as protein levels of HSL. In summary, our in vivo data confirm that Sct activates lipolysis through PKA to stimulate phosphorylation of HSL-660^ser^.

### Continuous Sct infusion does not increase circulating FFA levels

**Fig. 7.**
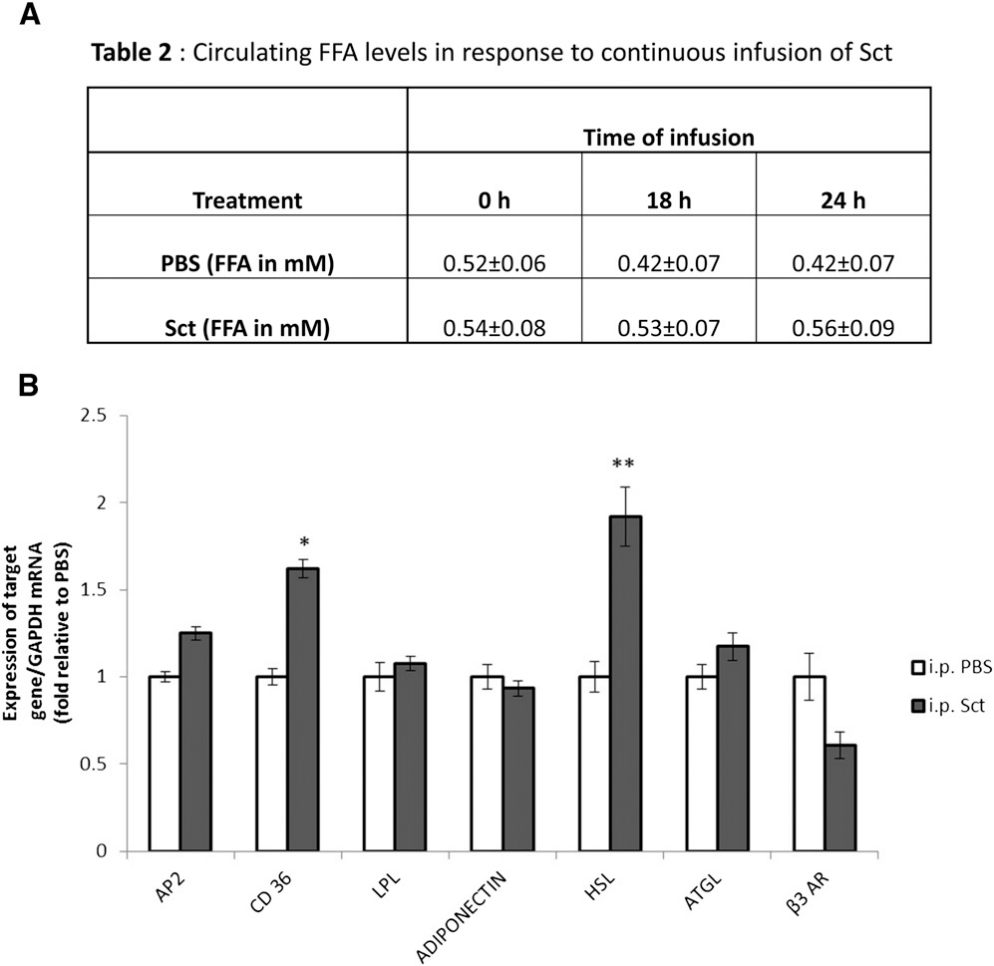
Continuous infusion of Sct does not increase circulating FFA levels. Mice (n = 5–6) that were fed ad libitum were infused with Sct at a concentration of 2.5 nmol/kg/day ip continuously through a mini-osmotic pump. A: Circulating FFA levels did not change significantly after continuous infusion of Sct. B: Transcript levels of a lipolytic gene (HSL) and a lipogenic gene (CD 36) were increased after continuous Sct infusion for 24 h. *P < 0.05, **P < 0.0005 from control.

Our in vivo and in vitro data consistently showed an acute lipolytic effect of Sct, therefore we sought to investigate its long-term effect on lipid metabolism by continuous infusion of Sct (2.5 nmol/kg/day) with a mini-osmotic pump. Sct infusion for 18 to 24 h was found to be unable to significantly change levels of circulating FFAs (**Fig. 7A**). When we measured expression changes of lipogenic and lipolytic genes, interestingly, a 24 h Sct infusion was found to elevate both lipolytic HSL as well as lipogenic gene cluster of differentiation 36 (CD 36) transcript levels (Fig. 7B). These data suggest a dual lipolytic and lipogenic role of long-term infusion of Sct that needs to be explored and clarified in future studies.

## DISCUSSION

**Fig. 8.**
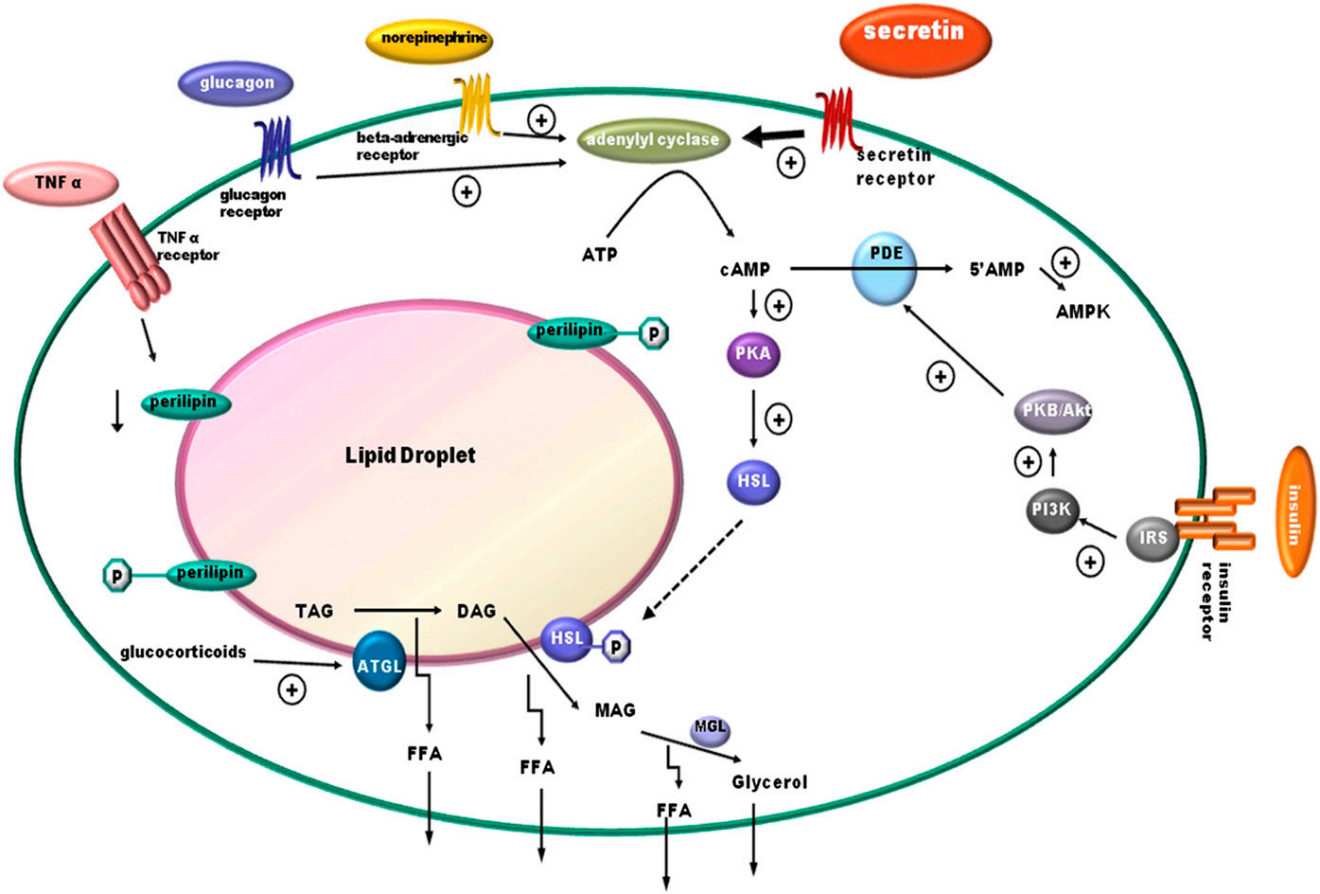
Working model summarizing the lipolytic effect of Sct. Sct binds SctR on the plasma membrane of adipocytes to activate the cAMP-dependent PKA. PKA then phosphorylates HSL at 660^ser^ leading to translocation of HSL from the cytosol to the phospholipid monolayer of the lipid droplet. The translocated HSL is responsible for the hydrolysis of stored triacylglycerol to release glycerol and FFAs. Norepinephrine (a catecholamine) and glucagon both activate the PKA pathway similar to Sct. TNF-α and glucocorticoids stimulate lipolysis by downregulating perilipin and enhancing the expression of ATGL, respectively. Insulin inhibits lipolysis by reducing the cAMP level through activation of PDE via the PI3K/Akt pathway. AMPK, AMP-activated protein kinase.

Catabolizing triacylglycerols stored in lipid droplets is a simple biochemical reaction; the process, however, is highly regulated and required for energy homeostasis (4–6). Catecholamine is known, so far, to be the key hormone involved in fasting-induced lipolysis, while glucocorticoids have also been shown to play a role (6). Among hormones that stimulate lipolysis, such as catecholamines, insulin, glucagon, growth hormone, and thyroid hormone (5, 6), the lipolytic effect of Sct remains elusive (23–28). Being a recently recognized anorectic hormone (20) with elevated plasma concentrations during fasting, as shown in our study and other studies (38–40), along with increased SctR expression in the epididymal adipose tissue during fasting (Fig. 1B), we therefore hypothesized the potential function of Sct in lipid metabolism. Consistent with this hypothesis, using Sct^−/−^ and SctR^−/−^ mouse models, we show here that Sct is able to stimulate dose- and time-dependent lipolysis in both in vitro adipocytes and in in vivo animals. This lipolytic effect is mediated by SctR expressed on the fat cells, which stimulates the cAMP-dependent protein kinase, PKA, which leads to phosphorylation of HSL, the rate-limiting enzyme for lipolysis (41), to bring about translocation of HSL to fat droplets for the activation lipolysis (**Fig. 8**). PKA is known to phosphorylate HSL at residues 565^ser^, 563^ser^, 660^ser^, and 659^ser^; where 565^ser^ is considered the basal phosphorylation site, while the other three serines are regulatory sites. Among them, phosphorylation at 660^ser^ was shown to be important for lipolysis because mutation of this residue resulted in a loss of enzymatic activity (42, 43). Consistent with this, we show that Sct is able to stimulate phosphorylation of HSL-660^ser^ in both in vitro and in vivo studies. Similar to catecholamines (42) and glucagon (44) that act via the PKA/HSL pathway to promote translocation of HSL from the cytosol to the phospholipid monolayer of the lipid droplet, we were also able to observe an accumulation of HSL surrounding the lipid droplet after Sct stimulation in Wt, but not in SctR^−/−^, adipocytes . It seems that catecholamines, glucagon, and Sct use the same intracellular pathway to stimulate lipolysis in the fat cells. A working model summarizing the molecular mechanism in the lipolytic effect of Sct is shown in Fig. 8.

While catecholamines are mainly responsible for acute lipolysis, other stimulants such as TNF-α and glucocorticoids have more chronic lipolytic effects (45). As shown in this study, Sct is able to stimulate an acute lipolytic response in vivo, evidenced by an increase in circulating FFA levels upon Sct injection (Fig. 5A). A 24 h continuous infusion of Sct through a mini-osmotic pump had no significant effect on levels of circulating FFAs, but could stimulate expressions of lipolytic HSL and lipogenic CD 36 genes, suggesting that effects of long-term Sct on lipid metabolism is different from its acute response. In fact, it has been reported that chronic elevated levels of Sct can increase FA uptake and triglyceride storage in fat cells, as well as upregulation of FA uptake genes including CD 36 in adipose cells in vitro (25). In addition, the expression of SctR has been found to be increased in the omental adipose tissue of obese individuals (46) and has been positively correlated with body mass index (25). This evidence, along with our findings, suggests a potential lipogenic role for long-term Sct, although this remains to be confirmed and the molecular mechanisms elucidated in future.

The balance between triacylglycerol hydrolysis and FFA esterification controls lipid homeostasis in our body. A slight alteration in these processes could therefore lead to metabolic disorders such as type 2 diabetes (47), hepatic steatosis (48), cardiovascular diseases (49), lipotoxicity (50), dyslipidemia (51), and certain types of cancer (52). In the case of obesity, higher levels of FFAs in the circulation due to changes in lipolytic rates result in the development of insulin resistance (53). In familial hyperlipidemia, decreased expression or function of HSL causes an impaired lipolytic function of catecholamines (54). It is therefore not surprising that proteins involved in lipid metabolism are targeted as pharmacological strategies against disorders such as obesity and metabolic syndrome (10, 55). This reinforces the relevance and importance of the need for more research on Sct and lipid metabolism. Our group and others have recently highlighted the importance of reevaluating the metabolic effects of Sct (20, 56). In this report, we have provided evidence to conclude that Sct has an acute lipolytic effect in vitro and in vivo. The relationship between lipid metabolism and Sct should warrant further studies in the future to provide an alternative therapeutic means to tackle various metabolic diseases.

## Supplementary Material

Supplemental Data
