# Supplementary material for: Lipolytic actions of secretin in mouse adipocytes
Source: J Lipid Res. 2014 Feb;55(2):190–200. doi: 10.1194/jlr.M038042 (PMC3886658; doi:10.1194/jlr.M038042)
Supplement: Supplemental Data [file supp_55_2_190__index.html]

Lipolytic Actions of Secretin in Mouse Adipocytes — Lipolytic actions of secretin in mouse adipocytes — Supplemental Data 

# Lipolytic actions of secretin in mouse adipocytes

## Supplemental Data

**Files in this Data Supplement:**

- Supplemental Fig. 1 - Comparison of changes in key lipolytic proteins by secretin and isoproterenol
