# Supplementary figures and images for: Lipolytic actions of secretin in mouse adipocytes
Source: J Lipid Res. 2014 Feb;55(2):190–200. doi: 10.1194/jlr.M038042 (PMC3886658; doi:10.1194/jlr.M038042)

**Figure 1**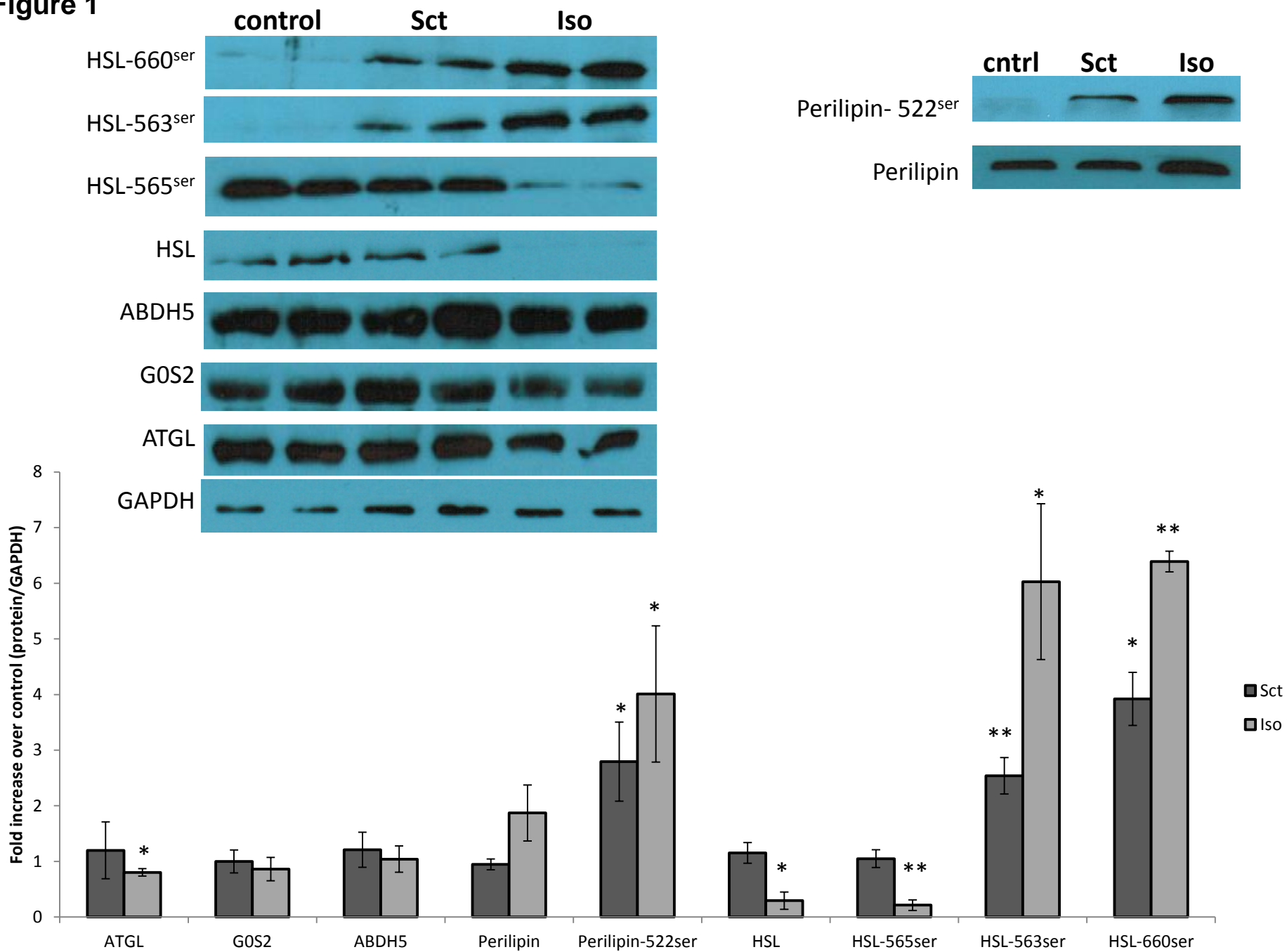

Supplement: Supplemental Data [file supp_M038042_jlr.M038042-1.pdf]
